# Multifocal Severe Coronary Artery Vasospasm Mistaken for Diffuse Atherosclerosis: A Case Report 

**DOI:** 10.1155/2010/202156

**Published:** 2010-09-01

**Authors:** Sarfraz Ahmed Nazir, Sheraz Nazir, Sanjay Kumar, Charles Ilsley

**Affiliations:** ^1^Department of Radiology, John Radcliffe Hospital, Headley Way, Oxford OX3 9DU, UK; ^2^Royal Brompton & Harefield NHS Foundation Trust, Hill End Road, Harefield, Middlesex UB9 6JH, UK

## Abstract

Spontaneous severe multivessel coronary artery vasospasm is a rare but important cause of morbidity. One-third of patients have normal coronary vasculature, and these pose a significant therapeutic dilemma as lack of clinical suspicion might potentially lead to unnecessary revascularization therapies. A patient with resting chest pain and ischaemic electrocardiography demonstrated severe coronary obstruction at catheter angiography. Preangioplasty further information highlighted spasm as the likely cause and the angiographic abnormalities resolved post intracoronary nitrate. This paper emphasises thorough history-taking and judicious use of nitrates during diagnostic coronary angiography in such patients. This may negate the need for more complex cardiac interventions.

## 1. Introduction

Spontaneous coronary artery vasospasm is defined as a transient total or near-total occlusion of a vessel, occurring in either a normal or diseased arterial segment, which is reversible with isosorbide dinitrate [1]. It is an important cause of morbidity, both in patients with proven coronary artery disease and in those with “a variant form of angina pectoris” as originally described by Prinzmetal et al. [2]. The latter typically is documented to present as chest pain occurring at rest, rather than as a consequence of physical exertion or emotional stress. Attacks of pain are observed to be cyclical and more common in younger patients. Although two-thirds of patients have concurrent atherosclerosis of a major coronary artery, this is often mild or out of proportion to the degree of symptoms. We report a case where multifocal coronary artery vasospasm was mistaken for obstructive diffuse atherosclerosis and almost resulted in unnecessary bypass grafting.

## 2. Case Presentation

A 50-year old Caucasian hypertensive exsmoker presented to the Harefield Hospital primary angioplasty service in March 2007 with an acute exacerbation of longstanding anginal chest pain at rest. 

Medical attention was not sought sooner, as the patient had assumed that her symptoms were the consequence of a putative chest infection for which she was taking antibiotics. Other daily medication included appropriate dosages of Clopidogrel, Dipyridamole, Ramipril and Nicorandil and sublingual GTN. The latter had only marginally improved symptoms. On clinical examination, heart rate and blood pressure were within normal limits, and auscultation revealed normal heart sounds and diffuse expiratory pulmonary wheeze.

**Figure 1:**
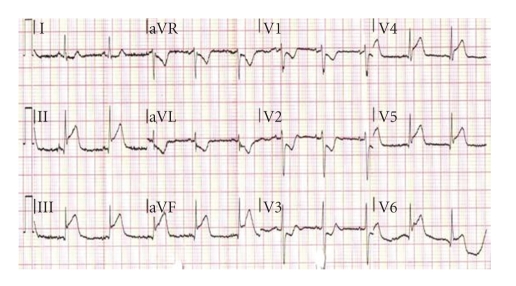
Presenting electrocardiogram demonstrating presumed ST-elevation myocardial infarction.

The serum creatine kinase (CK-MB) was normal. However, the combination of a mildly raised serum cardiac Troponin I (cTnI) of 1.1 ng/ml and the presenting ECG (Figure 1) necessitated the patient being prepared for primary angioplasty for inferolateral ST-elevation myocardial infarction. 

**Figure 2:**
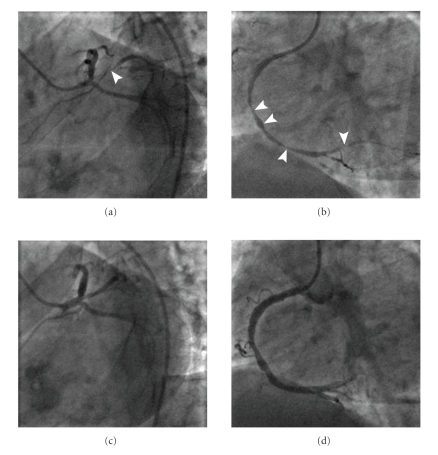
Selective Coronary Angiographic Images. Apparent stenoses in (a) intermediate and (b) right coronary arteries. Subsequent attenuation of lesions in the (c) intermediate and (d) right coronary arteries following administration of intra-coronary nitrate.

Selective coronary angiography demonstrated several obstructive stenoses in the right and intermediate coronary arteries (Figures 2(a)-2(b)). Complex intervention was contemplated, and the patient was offered angioplasty. At this stage, the patient fortuitously revealed that she was told she had unobstructed coronary arteries at angiography the previous year. Multivessel coronary artery vasospasm was now considered and intra-coronary nitroglycerin given. This induced relief of chest pain marked attenuation of all visible coronary stenoses (Figures 2(c)–2(d)) and resolution of ST segment elevation.

The patient was discharged with complete relief of symptoms and a normalization of the ECG on a regimen of a regular oral nitrate and high-dose calcium antagonist (Diltiazem). Thyroid function, vasoactive intestinal peptides, and urinary catecholamine levels were all normal. No primary cause for vasospasm was discovered.

## 3. Discussion

Coronary vasospasm is a transient abnormal contraction of an epicardial coronary artery which can instigate myocardial ischaemia [3]. The resultant spectrum of symptoms includes angina, myocardial infarction, arrhythmia, and sudden death. Although it can occur in vessel segments distressed by atherosclerosis, traditionally it has been often associated with variant or Prinzmetal's angina. First described in 1959, the latter is a syndrome where myocardial ischemia is induced by cyclical chest pain, usually occurring at rest in the early morning hours, and electrocardiographically most commonly associated with dynamic ST-segment elevation [2]. As in stable angina, the cardiac chest pain may be self-limiting or (usually) relieved by nitrate medication. Our case illustrates quite clearly that it is important that the diagnosis of vasospasm is made correctly and as early as possible. This may be hindered by the fact that the surface ECG can be entirely normal between attacks. Serum cardiac troponins also prove unreliable as they may or may not be raised and in cases such as ours may be actively misleading. The definitive diagnosis is made when angiographically demonstrated coronary artery vasoconstriction reverses with intravenous or intra-arterial nitroglycerin. 

Patients with variant angina tend to be younger in age than those with typical angina, and there is a relative higher incidence in younger women and in those with a systemic abnormality of vasomotor tone, such as sufferers of migraine headache and Raynaud's phenomenon [4, 5]. The absence of risk factors for atherosclerotic coronary artery disease suggests the diagnosis although cigarette smoking is a common risk factor for both clinical syndromes. In addition, racial heterogeneity probably exists in coronary artery vasomotor reactivity since coronary vasospasm and variant angina are more prevalent in the Japanese population than in Caucasians [6]. Patients from the Far East may exhibit a more diffuse abnormality of vasomotor tone, and angiography may show segmental or diffuse coronary artery spasm. In these populations, it appears that vasospasm is more readily considered in the differential diagnosis in the setting of acute chest pain. However, vasospasm in Caucasian patients raises treatment concerns. Beta-blockers are the first line therapy in patients with ischemic heart disease, angina pectoris, or acute coronary syndrome in the Western world but beta-blockade may aggravate coronary artery spasm without the administration of calcium channel blocker or nitrate. 

The chest pain associated with variant angina is commonly severe and may be accompanied by palpitations or syncope secondary to arrhythmia. It was originally believed that the pain occurred at rest and that exercise tolerance was characteristically normal in these individuals [2]. However, there is increasing evidence that the pain exhibits a circadian pattern, with most episodes occurring in the early hours of the morning. Some studies have shown that mild single-stage exercise is enough to induce variant angina in the early hours of the morning whereas even vigorous multistage exercise fails to do so in the afternoon [3].

The pathophysiological mechanism of vasospastic angina is thought to centre around a dysfunctional vascular endothelium [7]. Normally, the vascular tone of the endothelium is maintained by the interplay of vasorelaxing factors (such as nitric oxide and prostacyclin) and vasoconstrictors like endothelin-1. In the context of coronary vessels with a normal endothelium, acetylcholine(Ach) stimulates release of nitric oxide, which like external nitrates, should result in coronary vasodilatation. However, in those with an impaired endothelium, Ach fails to initiate release of nitric oxide and its absence results in direct action of Ach on the vessel and paradoxical vasoconstriction. In addition, nitric oxide also has antiplatelet effects and its lack compounds the problem by the creation of a prothrombotic environment. 

The most common cause of deranged endothelial function is in the setting of atherosclerosis. Historically, the highest risk zones for acute coronary artery occlusion are the proximal thirds of the vessels, and most incidences of focal vasospasm occur at these regions [8]. There is relatively greater plaque burden in the proximal thirds of coronary arteries, and so it comes as no great surprise that vasospasm is most prevalent at the site of coronary atheromata. This may seem at odds with the findings in our case, where the focal coronary spasm responded to intravenous nitrate and the arteries were subsequently found to *appear* normal. However, it is reported that vasospasm in coronary vessels that seem normal on angiography may have at least minimal atherosclerotic change not detected by coronary angiography but detected by more sensitive techniques such as intravascular ultrasonography [9]. 

Pharmacological first-line management of coronary vasospasm relies on drugs that promote direct vasodilatory effects on the coronary vasculature. The most frequently used are calcium channel blockers, alone or in combination therapy with long-acting nitrates. By working synergistically to abolish or limit the vasoconstricting effects of the damaged endothelium, they prevent vasospasm-induced ischaemia. For those that are intolerant or refractory to these medications, the therapeutic options include administration of selective alpha-adrenoceptor antagonists (prazosin) [10], drugs that promote vasodilatation via the activation of potassium channels (nicorandil) [11], or in the case of diabetics, troglitazone may be efficacious [12]. Revascularisation therapies (percutaneous coronary artery intervention or bypass grafting) have to be considered in the 20% of patients that do not respond to medical treatment. One should bear in mind however, that in up to one-third of these patients coronary vasospasm can still be induced at arterial segments not covered by the intervention [13, 14]. In patients with known atherosclerotic coronary artery disease, meticulous attention should be paid to risk factor modification. Certainly the long-term survival and incidence of acute myocardial infarction have been shown to depend on the degree of underlying coronary artery disease. Specifically, there has been shown to be a correlation between the extent of fixed atherosclerotic thrombo-occlusive disease, the degree of ST-elevation during acute episodes, the frequency and duration of attacks, and the magnitude of ventricular dysfunction [15].

## 4. Conclusion

Coronary artery vasospasm can cause a transient, abrupt, and marked decrease in the diameter of an epicardial coronary artery. Various aetiological mechanisms have been implicated which are all probably related to an exaggerated contractile response of the vascular smooth muscle in the affected coronary vessels. Spasm usually affects atherosclerosis-afflicted segments in the coronary vascular tree, but as in our case it may also occur in seemingly angiographically normal arteries, albeit that subclinical atherosclerotic change in such arteries may be detected by more sensitive techniques such as intravascular ultrasonography.

This paper illustrates the clinical importance of multivessel coronary artery vasospasm in mimicking the angiographic appearances of diffuse coronary atherosclerosis. It exemplifies the importance of a detailed clinical history and listening to the patient, because it may suggest the role of dynamic vasomotor components. Administering intra-coronary nitrate before embarking on angioplasty resulted in the correct diagnosis, complete resolution of symptoms, and the initiation of appropriate long-term therapy. Perhaps more importantly, it prevented an unnecessary invasive coronary procedure. Vasospasm should therefore always be included in the differential diagnosis in suggestive presentations of acute coronary syndrome. We therefore suggest the use of intra-coronary nitrate to be mandatory in all patients with coronary stenosis or obstructions before considering angioplasty to avoid the potential pitfall of inappropriate coronary intervention.

## References

[1] Ozaki Y, Keane D, Serruys PW (1995). Progression and regression of coronary stenosis in the long-term follow- up of vasospastic angina. *Circulation*.

[2] Prinzmetal M, Kennamer R, Merliss R, Wada T, Bor N (1959). Angina pectoris I. A variant form of angina pectoris. Preliminary report. *The American Journal of Medicine*.

[3] Yasue H, Omote S, Takizawa A (1979). Circadian variation of exercise capacity in patients with Prinzmetal’s variant angina: role of exercise-induced coronary arterial spasm. *Circulation*.

[4] Beltrame JF, Sasayama S, Maseri A (1999). Racial heterogeneity in coronary artery vasomotor reactivity: differences between Japanese and caucasian patients. *Journal of the American College of Cardiology*.

[5] Keller KB, Lemberg L (2004). Prinzmetal’s angina. *American Journal of Critical Care*.

[6] Miwa K, Fujita M, Sasayama S (2005). Recent insights into the mechanisms, predisposing factors, and racial differences of coronary vasospasm. *Heart and Vessels*.

[7] Sztajzel J, Mach F, Righetti A (2000). Role of the vascular endothelium in patients with angina pectoris or acute myocardial infarction with normal coronary arteries. *Postgraduate Medical Journal*.

[8] Koizumi T, Yokoyama M, Namikawa S (2006). Location of focal vasospasm provoked by ergonovine maleate within coronary arteries in patients with vasospastic angina pectoris. *American Journal of Cardiology*.

[9] Yamaguchi M, Miyatake K, Tamai J, Nakatani S, Koyama J, Nissen SE (1994). Intravascular ultrasound detection of atherosclerosis at the site of focal vasospasm in angiographically normal or minimally narrowed coronary segments. *Journal of the American College of Cardiology*.

[10] Tzivoni D, Keren A, Benhorin J (1983). Prazosin therapy for refractory variant angina. *American Heart Journal*.

[11] Kaski JC (1995). Management of vasospastic angina—role of nicorandil. *Cardiovascular Drugs and Therapy*.

[12] Murakami T, Mizuno S, Ohsato K (1999). Effects of *Troglitazone* on frequency of coronary vasospastic-induced angina pectoris in patients with diabetes mellitus. *American Journal of Cardiology*.

[13] Rodgers GP, Raizner AE, Cromeens DM (1989). Coronary spasm induced by stent implantation. *Journal of the American College of Cardiology*.

[14] Rabinowitz A, Dodek A, Carere RG, Webb JG (1996). Stenting for treatment of coronary vasospasm. *Catheterization and Cardiovascular Diagnosis*.

[15] Walling A, Waters DD, Miller DD, Roy D, Pelletier GB, Theroux P (1987). Long-term prognosis of patients with variant angina. *Circulation*.

